# Evaluation of new and old biomarkers in dogs with degenerative mitral valve disease

**DOI:** 10.1186/s12917-022-03343-z

**Published:** 2022-07-02

**Authors:** Stephanie Klein, Ingo Nolte, José Luis Granados-Soler, Philipp Lietz, Maximiliane Sehn, Jonathan Friedemann Raue, Karl Rohn, Eva-Maria Packeiser, Jan-Peter Bach

**Affiliations:** 1 Clinic for Small Animals, University of Veterinary Medicine Hannover, Foundation, Hannover, Germany; 2 Institute for Biometry, Epidemiology and Information Processing, WHO Collaborating Centre for Research and Training for Health at the Human-Animal-Environment Interface, University of Veterinary Medicine Hannover, Hannover, Germany

**Keywords:** Biomarker, DMVD, Dog, Galectin-3, ST2

## Abstract

**Background:**

Dogs with degenerative mitral valve disease are commonly presented to small animal clinicians. Diagnosis, clinical staging, and therapeutic design are based on a combination of clinical examination, radiography, and echocardiography. To support diagnosis and clinical monitoring, a multi-marker-based approach would be conceivable. The aim of this study was to investigate the suitability of Galectin-3 and interleukin-1 receptor-like 1 protein (ST2) in dogs with degenerative mitral valve disease in accordance with N-terminal-prohormone-B-type natriuretic peptide (NT-proBNP) and cardiac troponin I (cTnI). For this purpose, serum concentrations of Galectin-3 and ST2 of 64 dogs with different stages of mitral valve disease and 21 dogs without cardiac disease were analyzed at the first examination and six months later. Echocardiography, blood cell count and clinical chemistry were performed and established biomarkers NT-proBNP and cTnI were measured additionally. Differences in the biomarker concentrations between all groups at both timepoints and the change in biomarker concentrations from first to second evaluation was investigated. Furthermore, correlations of each biomarker, between biomarkers and echocardiographic measurements, were calculated. Finally, the receiver-operating characteristic curve and the area under the curve analysis were performed to differentiate between disease stages and controls.

**Results:**

Serum concentrations of Galectin-3 and ST2 were not statistically different between canine patients in the respective stages of mitral valve disease or in comparison to dogs in the control group at any timepoint. A significant increase in ST2 concentrations from the baseline to the follow-up examination was observed in dogs classified as stage B1 and the control group. The concentrations of NT-proBNP and cTnI in stage C dogs were significantly increased in comparison to the other groups.

**Conclusions:**

In this study, no relation between Galectin-3 and ST2 levels to the presence or stage of mitral valve disease could be detected. Nevertheless, considering the increase in ST2 concentrations from the first to second measurement, its value on monitoring disease progress could be feasible. In agreement with previous studies, NT-proBNP and cTnI have once more proven their utility in assessing disease severity. The approach of examining new cardiac biomarkers in dogs is still worth pursuing.

## Introduction

The therapeutic approach to dogs with degenerative mitral valve disease (DMVD) is based on disease severity [1–3], which is often staged according to the latest consensus guidelines published by the American College of Veterinary Internal Medicine (ACVIM) [4]. ACVIM staging is based on the combined findings of anamnesis, clinical examination, echocardiography, and radiography. Reported limitations for measuring and interpreting echocardiograms and thoracic radiographs are not just related to the various sizes and forms of dog breeds but are also susceptible to different techniques, equipment, patient positioning, and individual performance of the examinations and measurements [5–9]. Moreover, echocardiography, which is of major importance for diagnosing and monitoring DMVD, as well as for therapeutic decision-making and therapy adjustment, requires special training and equipment. As the location of veterinary cardiologists is mostly linked to larger clinics, this limits the access for some dog owners. Therefore, facilitating the identification and monitoring of cardiac diseases through the investigation of blood-based biomarkers is a promising approach. Well-functioning biomarkers are objective and broadly applicable due to their ease of use [10].

Cardiac biomarkers, such as N-terminal-prohormone-B-type natriuretic peptide (NT-proBNP) and cardiac Troponin I (cTnI) have gained importance in small animal cardiology [11–16]. The main stimulus for the secretion of NT-proBNP is myocardial stretch due to volume overload [17, 18]. CTnI is released into circulation as a result of myocardial cell injury [16, 19, 20]. However, despite their reported predictive value, they are mainly used as supplementary diagnostic techniques in canine DMVD [21]. Furthermore, reported limitations include kidney disease influence on NT-proBNP measurements and cTnI correlation with age [15, 22]. Considering the multisystemic character of heart failure (HF), a multimarker strategy has gained much attention in human cardiology, where many different cardiac biomarkers are available [23–25]. The measurement of multiple biomarkers in human cardiac patients has been proven to be superior in diagnosis, prognosis, and risk stratification [26–31]. In contrast, the identification of reliable predictors of DMVD progression and adverse cardiac outcomes, that might be useful for the development of a multimarker panel, is still needed in dogs.

Studies in human cardiac patients support the evaluation of Galectin-3 and interleukin-1 receptor-like 1 protein (ST2) as strong predictors of mortality and adverse outcomes [28, 32–36]. Furthermore, both ST2 and Galectin-3 are involved in pathophysiologic processes of heart disease in humans and are categorized as markers of myocardial fibrosis and cardiac remodeling. Galectin-3 is released from activated macrophages and plays a physiological role in tissue repair. However, its overexpression promotes cardiac fibrosis [37, 38]. The protein products of the *ST2* gene include the transmembrane (ST2L) and the soluble isoform (sST2), which are upregulated in and released from endothelial cells, and to a minor degree from cardiomyocytes and fibroblasts due to biomechanical strain [39–43]. Likewise, the functional ligand of ST2 (interleukin-33; IL-33) is released from cardiac fibroblasts in response to damage, and its interaction with ST2L mediates a cardioprotective mechanism that counteracts cardiac hypertrophy and fibrosis, reduces apoptosis, and improves myocardial function [39, 40, 44]. When IL-33 is instead bound to sST2 (hereinafter referred to as ST2), the decoy-receptor hinders IL-33 in binding to ST2L, inhibiting the IL33/ST2L signaling and consequently the antihypertrophic mechanism [39, 41]. In humans, ST2 is superior to Galectin-3 in predicting adverse outcomes [34] and is not influenced by comorbidities like chronic kidney disease [45], age, or gender, and is subject to less biological variability [34, 46]. Literature in veterinary cardiology studying these two biomarkers in dogs with heart disease is still scarce. On the one hand, studies evaluating ST2 concentrations, have not yet been able to report increased ST2 values in dogs with heart disease [47, 48]. On the other hand, studies investigating circulating Galectin-3 concentrations and its expression in heart tissue have already shown some promising results [49, 50].

This study aimed to investigate the applicability of Galectin-3 and ST2 to differentiate between healthy dogs and canine patients suffering from DMVD across different clinical stages, and to evaluate the suitability of these biomarkers to monitor disease progression over time. Consequently, serum concentrations of the novel biomarkers were measured at two different timepoints and correlated for different clinical stages and in comparison to healthy dogs. Furthermore, to detect additional correlations and agreement with established biomarkers, NT-proBNP and cTnI were also measured.

## Material and methods

### Animals

This study was prospectively conducted after approvement of the responsible ethical committee (Lower Saxony State Office for Consumer Protection and Food safety [LAVES], 33.8–42,502-05-18A321). Furthermore, written informed consent from the owners of all participating dogs was obtained.

Ninety-two client-owned dogs of various breeds with DMVD, which were presented to the Clinic for Small Animals of the University of Veterinary Medicine Hannover, Hannover, Germany were prospectively enrolled in the study. Of these 92 animals, 85 were considered for final analysis, as seven dogs were excluded due to other concurrent diseases. The baseline characteristics including demographics, renal values, echocardiographic measurements, and heart medication are shown in Table 1. Dogs with DMVD (*n* = 64) were grouped according to ACVIM recommendations, including 41 (64.1%) dogs in stage B1, 9 (14.1%) in stage B2, and 14 (21.9%) in stage C. Patients in stage C displayed single or multiple symptoms, including coughing (10/14), exercise intolerance (5/14), dyspnea (6/14), tachypnea (4/14), and syncope (2/14). No restrictions were made concerning previously prescribed heart medications (find the applied heart medication in Table 1). Dogs with DMVD (*n* = 64) ranged in age from 4.4 to 16.6 years, with a mean of 11.2 (± 2.6) years and a median weight of 9.8 kg (7.6—15.1 kg). Patients’ gender distribution was balanced with 31 females (48.4%), of which 26 (83.9%) were spayed, and 33 males (51.6%), of which 19 (57.6%) were neutered. DMVD patients included in this study represented several breeds, among which mixed-breed dogs were the most common (*n* = 18; 28.1%), followed by Dachshund (*n* = 11; 17.2%), Cavalier King Charles Spaniel (CKCS) (*n* = 4; 6.3%), and Beagle (*n* = 3; 4.7%). The control group (*n* = 21) included dogs without concurrent systemic or cardiac disease detected by clinical, echocardiographic, and blood examination. Healthy controls ranged in age from 3.2 to 12.8 years, with a mean of 8.7 (± 2.3) years and a median weight of 7.8 kg (6—10 kg). The control group consisted of 10 females (47.6%), of which 4 (40%) were spayed, and 11 males (52.4%), of which 9 (81.8%) were neutered. Most represented breeds were Dachshund (*n* = 5; 23.8%) and mixed-breed dogs (*n* = 3; 14.3%).

**Table 1:** Baseline characteristics of healthy controls and DMVD patients included in the study

Parameters	Control *n* = 21	Stage B1 *n* = 41	Stage B2 *n* = 9	Stage C *n* = 14
***Demographics***				
** Breed (Mixed breed/Dachshund/CKCS/Beagle/Others)**	(3/5/0/2/11)	(14/7/3/3/14)	(0/3/0/0/6)	(4/1/1/0/8)
** ªAge (years)**	8.7 (± 2.3)	10.8 (± 2.5)*	11.1 (± 2.6)	12.5 (± 2.6)*
	8.3 (7.8–10.7)	11.2 (9.8–12.8)	10.7 (10.5–12)	12.7 (11.8–13.4)
** ªWeight (kg)**	9.3 (± 4.7)	12.5 (± 6.4)	12.8 (± 7.2)	9.2 (± 5.2)
	7.8 (6–10)	11 (8–16.6)	9.8 (7.9–19.5)	7.5 (6.4–11.1)
** Sex (f/fs/m/mn)**	(6/4/2/9)	(2/18/6/15)	(3/1/3/2)	(0/7/5/2)
***Laboratory variables***				
** ªBUN (mg/dL)**	38.7 (± 17.9)***	35 (± 11.3)***	49.9 (± 26.7)	58.9 (± 58.9)
	34 (28.5–39.5)	33 (26.5–44)	37 (26–73)	55.5 (37–76)
** ªCREA (mg/dL)**	0.78 (± 0.18)	0.79 (± 0.21)	0.84 (± 0.37)	0.79 (± 0.2)
	0.71 (0.66–0.86)	0.79 (0.65–0.91)	0.97 (0.59–1.02)	0.77 (0.66–0.94)
***Echocardiographic measurements***				
** ªLA/Ao**	1.24 (± 0.18)	1.39 (± 0.22)*	2.14 (± 0.36)*^/^**	2.31 (± 0.57)*^/^**
	1.23 (1.12–1.32)	1.42 (1.25–1.57)	2.04 (1.89–2.45)	2.22 (1.79–2.69)
** ªLVIDDn**	1.49 (± 0.15)	1.6 (± 0.21)	1.88 (± 0.26)*^/^**	2.04 (± 0.32)*^/^**
	1.48 (1.39–1.58)	1.57 (1.45–1.74)	1.97 (1.77–2.02)	1.99 (1.76–2.43)
** Reflux (0/1/2/3)**	(21/0/0/0)	(0/23/13/5)	(0/1/2/6)	(0/0/2/12)
***Heart medication***^***b***^				
** ACEI (yes/no)**	(0/21)	(2/40)	(4/8)	(6/10)
** Pimobendan (yes/no)**	(0/21)	(4/37)	(6/3)	(7/5)
** Loop Diuretics (yes/no)**	(0/21)	(0/41)	(0/9)	(3/11)
** Spironolactone (yes/no)**	(0/21)	(1/40)	(3/6)	(3/11)

ªThe first row of these parameters shows the mean (± standard derivation) values, the second displays the median (interquartile range); f female; fs, female spayed; m, male; and mn, male neutered; ^b^ Patients may be treated with a combination of the mentioned medical agents so that the number might exceed that of the total group size, *ACEI* Angiotensin-Converting Enzyme Inhibitor, *BUN* Blood Urea Nitrogen, *CKCS* Cavalier King Charles Spaniel, *CREA* Creatinine, *LA/Ao* Ratio left atrium to aortic root, *LVIDDn* Left ventricle internal diastolic diameter normalized, Reflux size: 0, < 15%; 1, 15–30%; 2, 30–50%; and 3 > 50%;

^*^ significantly different from control; ** significantly different from stage B1; *** significantly different from stage C

### Diagnostic examinations and classification of enrolled patients

All dogs underwent a complete physical examination and received a laboratory workup, which included a total blood count and clinical chemistry. If clinically indicated, electrolytes were also measured. Patient eligibility criteria included the presence of a left-sided systolic heart murmur. A detailed clinical history was recorded with concern being taken to present and/or past symptoms and treatment regime.

To diagnose and stage DMVD patients and to confirm the absence of cardiac disease in the control group, an echocardiographic examination with a simultaneous electrocardiogram (Vivid E7 and E9, GE Healthcare GmbH, Solingen, Germany) was performed in all animals included in the study. Furthermore, mild tricuspid and pulmonary valve insufficiencies were tolerated, whereas other structural cardiac abnormalities led to the exclusion of dogs. Mitral regurgitation and its direction were visualized with color-Doppler. The size of the regurgitation jet was determined by estimating its maximal expansion and setting it in relation to the left atrium area [51]. Following a previous study by Giraut et al. (2019), mitral regurgitation was considered clinically non-significant when the regurgitant jet area to left atrium area ratio was less than 15%, and dogs with such minor regurgitation were eligible for inclusion into the healthy group if no cardiac murmur was perceivable on auscultation. Larger regurgitations were classified as mild if the jet area to left atrium ratio was between 15–30%, as moderate between 30–50%, and as severe when greater than 50% [52]. M-mode measurements to determine heart enlargement were obtained from long-axis (ventricle) and short-axis (atrium) views. The size of the left ventricle was determined by the left ventricular internal end-diastolic diameter normalized for the dogs’ body weight (LVIDDn) as described by Cornell et al. (2004) [53]. The size of the left atrium was assessed by measuring the diameters of the left atrium and aorta at heart base level and calculating their ratio (LA/Ao) using the method established by Hansson et al. (2002) [54]. For the calculations, the averaged measurements of both parameters in the short-axis of three cardiac cycles were used.

After clinical and echocardiographic examination, patients were categorized as stage B1, B2 or C according to the criteria in the ACVIM consensus statement for DMVD [4]. Accordingly, dogs with mitral regurgitation and absent cardiomegaly were classified as stage B1, dogs with cardiomegaly were classified as stage B2. Dogs with cardiomegaly and present or a previous phase of symptoms were classified as stage C. All these examinations were re-evaluated after six months at a follow-up examination for both patients and controls, and patients were reclassified accordingly if disease progression was detected.

### Measurements of cardiac biomarkers

Venous blood samples were collected into serum and EDTA- tubes at each examination and were centrifuged within 60 min after collection for 10 min at 1000 × g. After the separation, serum and plasma samples were stored at -80 °C until measured collectively.

Serum concentrations of both ST2 and Galectin-3 were determined using the enzyme-linked immunosorbent assay (ELISA) technique. Pre-experiments were carried out to determine in which range the concentrations would have to be expected. For the pre-experiments, competitive ELISAs (BlueGene Biotech CO., LTD., Shanghai, China) were used. However, due to low performance and measurements mostly below the detection limit, further investigations were performed with other research-use quantitative-specific sandwich-type ELISAs for each biomarker. Consequently, ST2 was determined with the “Canine Interleukin 33 Receptor (ST2) ELISA Kit” (MyBioSource, Inc., San Diego, CA, USA) with a detection range of 0.625–20 ng/mL. Undiluted samples were used for the measurements and values below the limit of quantification (LOQ) (0.625 ng/mL) were set to LOQ/2 for calculations. For Galectin-3 measurements, the “RayBio® Canine Galectin-3 ELISA Kit” (RayBiotech, Inc., Peachtree Corners, GA, USA) with a detection range of 2–500 pg/mL was used. As this kit required diluted samples, a few dilution series were performed to determine which dilution factor was most appropriate; accordingly, 1:30 was used as the initial dilution. If the measurements resulted in values below or above the detection range, the dilution was adjusted to either 1:10 or 1:70. All experiments were performed following the user manuals and all measurements were run in duplicate. Further data analysis was conducted with the mean of the two measurements. The readings for optical density were performed with a multi-mode microplate reader (Synergy TM 2, BioTek® Instruments, Inc., Winooski, VT, USA) with a wavelength of 450 nm. For calculating inter-, and intra-assay coefficients of variation (CV), a control serum sample was added on each plate four times in duplicate.

Plasma and serum samples for measuring NT-proBNP and cTnI were shipped on ice to IDEXX-Laboratories (Ludwigsburg, Germany). The measurements were performed with the established Cardiopet® proBNP and high-sensitivity Troponin I test.

### Statistical methods

Statistical analyses were performed using SAS 9.4 (SAS Institute Inc., Cary, NC, USA) in conjunction with GraphPad Prism version 8.0.0 (GraphPad, San Diego, CA, USA). The level of significance was set at a *p*-value < 0.05.

All parameters were tested for normal distribution with the Shapiro–Wilk test. According to distribution, data are presented as mean (± standard deviation [SD]) or median (interquartile range [IQR]). Because of rejection of the normal distribution assumption for most continuous variables, nonparametric procedures were used. Differences in the biomarker concentrations between all groups at both timepoints were analyzed using the Kruskal–Wallis test with the post-hoc Dwass, Steel, Critchlow-Fligner-test for multiple pairwise comparisons, adjusting for the experiment-wise error rate. Due to insufficient sample quantity in some cases, not all calculations could be performed with the original group size. The change in biomarker concentrations from the baseline to the follow-up examination was analyzed with the Wilcoxon Signed Rank Test for paired sample. Spearman’s rank-order correlation coefficient was calculated to describe the relationships between biomarkers and echocardiographic measurements. The null hypothesis in the correlation test is *r *= 0. If *p* value < 0.05, this hypothesis is rejected and a significant correlation is assumed. The strength of correlation was rated as fair when < 0.5, as moderate when 0.5—0.6, as very strong when 0.8—0.9, and perfect when 1 [55]. Finally, to determine the diagnostic ability of each biomarker to differentiate patients according to clinical staging, as well as patients from controls, and to define optimal discrimination limits, the receiver-operating characteristic (ROC) curve and area under the curve (AUC) analysis were performed. The results of the AUC were rated as less accurate when 0.5 < AUC < 0.7, moderately accurate when 0.7 < AUC < 0.9, highly accurate when 0.9 < AUC < 1, and perfect when AUC = 1 [56]. Due to the sample sizes and possible combination of medication, the influence of the medication on the biomarker measurements was not analyzed statistically.

## Results

### Baseline examination

Age was normally distributed, whereas weight was not. Fifty-eight dogs met the inclusion criteria representing 64 DMVD patients and 21 healthy controls. Although care was taken to match the demographics of the control group with those of the DMVD patients, the controls were significantly younger (*p* = 0.0001).

### Follow-up examination

As not all dogs were presented for the routine follow-up examination and some DMVD patients were reclassified into a different stage due to disease progression, group sizes at follow-up differed from those at the baseline examination (Table 2). Three dogs of 21 in the control group were not presented for follow-up evaluation due to unknown reasons. Of 64 dogs with DMVD, 52 remained in the study after six months. Among these 12 patients that were lost to follow-up, 6 were not presented due to unknown reasons, and 6 (all stage C) died before the completion of the study period. Thirty-eight of 41 dogs initially classified as stage B1 were presented at the follow-up examination, of which 5 progressed to stage B2, resulting in a group size of *n* = 33 for stage B1. Among patients classified as stage B2 (*n* = 9) during the baseline examination, one was lost to follow-up and one progressed to stage C. Furthermore, the reclassification of the 5 patients that progressed from stage B1 to stage B2 resulted in a B2 group size of *n* = 12 at the second evaluation. Finally, of 14 patients initially classified as stage C, 6 died before the end of the study period. Five of these dogs died or were euthanized due to cardiac disease. Two dogs were lost to follow-up and one dog progressed from stage B2 to stage C. Thus, stage C included *n* = 7 patients at the follow-up examination.

**Table 2:** Group compositions at baseline examination and reclassification at follow-up examination

Baseline		Follow-up	
**Stage B1**	*n* = 41	**Stage B1**	*n *= 33
		**Stage B2**	*n* = 5
**Stage B2**	*n *= 9	**Stage B2**	*n* = 7
		**Stage C**	*n* = 1
**Stage C**	*n* = 14	**Stage C**	*n* = 6
		**Dead**	*n* = 4

This table displays the classification of the DMVD patients at baseline examination and reclassification at follow-up examination. The difference in number from baseline to follow-up are the dogs that were not presented at second evaluation

### Galectin-3 and ST2 measurements

**Fig. 1:**
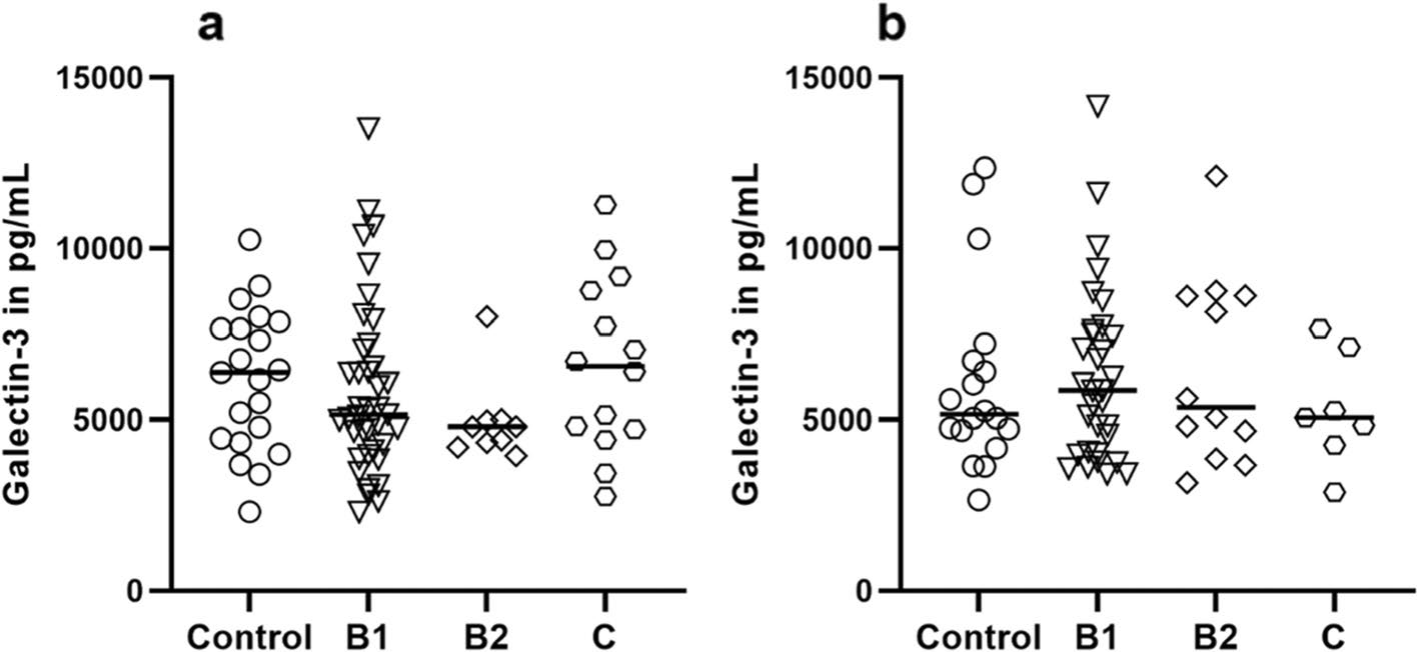
Galectin-3 concentrations in pg/mL for each dog in the respective groups at **a**) baseline and **b**) follow-up examination, median marked as a bar. No significant differences between the groups could be detected

Galectin-3 concentrations (Table 3) in all evaluated dogs with DMVD (*n* = 62) ranged from 2297.32 to 13,508.02 pg/mL (median [IQR], 5069.60 [4334.51–7049.37] pg/mL) at the first measurement, and remained similar (*p* = 0.5465) after six months (*n* = 51; median [IQR] 5528.56 [4055.5–7655.21] pg/mL) with intra- and inter-assay CV of 6.89% and 20.55%, respectively. In the control group (*n* = 21), Galectin-3 concentrations ranged from 2305.2 to 10,255.38 pg/mL (median [IQR], 6380.50 [4462.49–7656.29]) at baseline and 2650.32 to 12,351.73 pg/mL (median [IQR], 5152.05 [4681.11–6713.43]) at follow-up examination (*n* = 18) (*p* = 0.5262). No statistical differences between patients and controls or across different clinical stages were detected at any evaluation performed (Fig. 1a and b).

**Table 3:** Serum Galectin-3 concentrations in pg/mL for the respective groups at baseline and follow-up examination

Groups	Baseline (*n*; median [IQR])	Follow-up (*n*; median [IQR])	*P*-value
Control	21; 6380.5 [4462.49–7656.29]	18; 5152.05 [4681.11–6713.43]	NS
Stage B1	39; 5140.91 [4094.13–7047.52]	32; 5851.08 [4033.94–7557.08]	NS
Stage B2	9; 4791.34 [4334.51–4969.2]	12; 5352.87 [4261.51–8612.47]	NS
Stage C	14; 6556.58 [4713.07–8767.38]	7; 5056.82 [4256.65–7119.38]	NS

*IQR* Interquartile range, *NS* Non-significant. The *p*-value denotes the probability of the change in Galectin-3 concentrations from baseline to follow-up examination for each group. Data are presented as median (interquartile range)

**Fig. 2:**
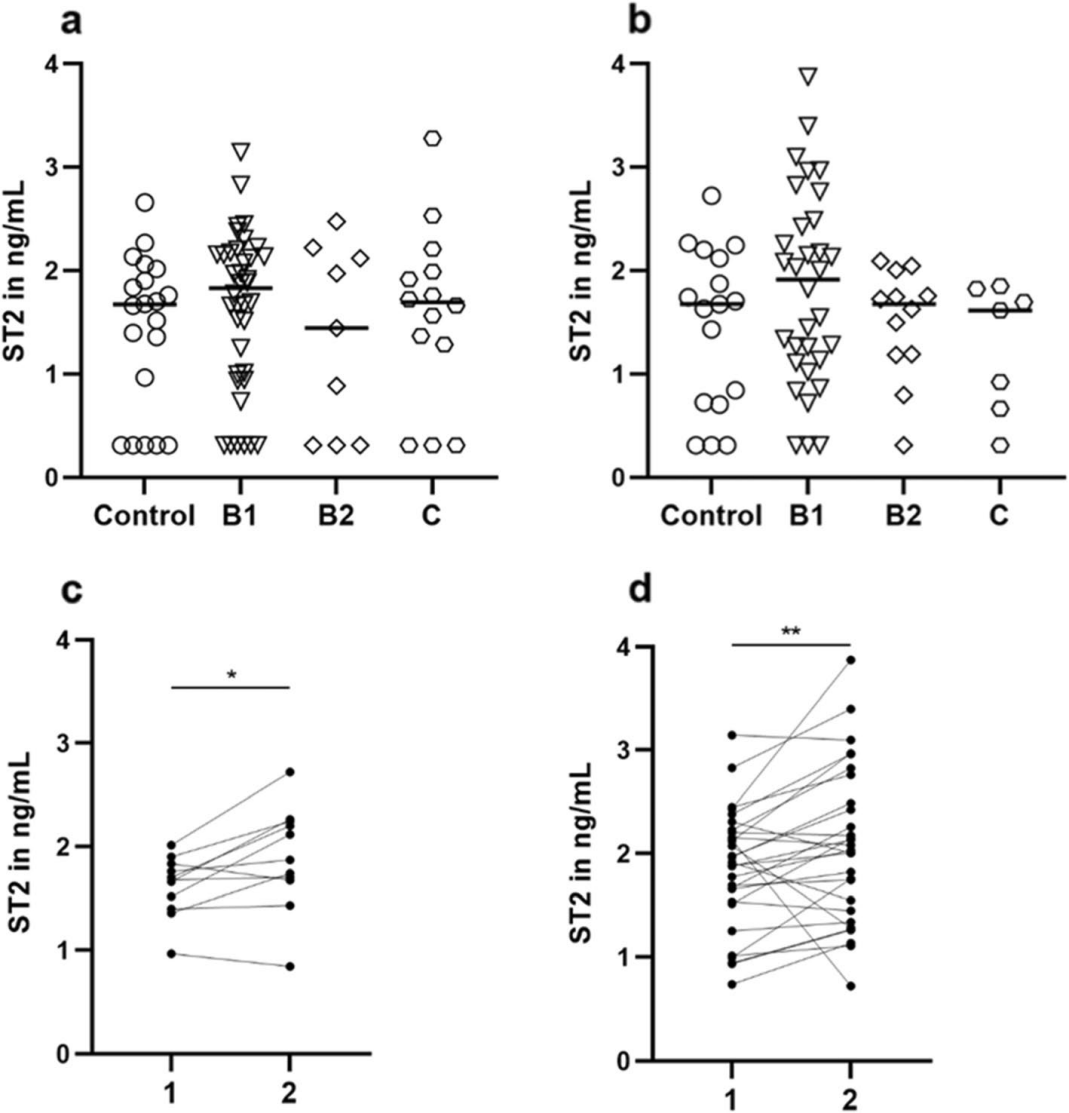
ST2 concentrations in ng/mL for each dog in the respective groups at **a**) baseline and **b**) follow-up examination, median marked as a bar. No significant differences between the groups could be detected. Significant changes in ST2 concentrations from baseline (**1**) to follow-up (**2**) examination occurred in **c**) control group and **d**) initially in stage B1 patients. * *p* < 0.05, ** *p* < 0.001

Initial ST2 concentrations (Table 4) in all evaluated dogs with DMVD (*n* = 61) ranged from 0.31 to 3.28 (median [IQR] 1.72 [0.94–2.15]) ng/mL, remained similar (*p* = 0.9022) after six months follow-up (*n* = 51; 1.70 [1.11–2.13] ng/mL), and were not statistically different from controls or between different stages at any comparison (Fig. 2a and b). The ST2 concentrations in the control group ranged from 0.31 to 2.69 ng/mL (median [IQR], 1.67 [0.64–1.96]) at baseline (*n* = 20) and 0.31 to 2.72 ng/mL (median [IQR], 1.67 [0.72–2.12] at follow-up examination (*n* = 17) (*p* = 0.8064). Furthermore, the intra- and inter-assay CV were 11.83% and 16.64%, respectively. Specifically, a significant increase in ST2 concentrations from the baseline to follow-up examination was observed in dogs classified as stage B1 (*p *= 0.0007) and controls (*p* = 0.041), see Fig. 2c and d. This was not the case for stages B2 and C. Of note, of the 5 stage B1 dogs that progressed to B2, 4 dogs showed increased ST2 concentrations at the second measurement (the baseline sample was not available for the fifth patient due to insufficient serum material). Of these 4 dogs with increased ST2 concentrations, 2 dogs did not receive medication. In the other 2 dogs, medical treatment was initiated before the second measurement. One patient that progressed from stage B2 to stage C was put on medication right after the first investigation. ST2 values increased at follow-up.

**Table 4:** ST2 concentrations in ng/mL for the respective groups at baseline and follow-up examination

Groups	Baseline (*n*; median [IQR])	Follow-up (*n*; median [IQR])	*P*-value
Control	20; 1.67 [0.64–1.96]	17; 1.68 [0.72–2.12]	0.0413*
Stage B1	38; 1.87 [0.99–2.16]	32; 1.91 [1.12–2.46]	0.0007**
Stage B2	9; 1.44 [0.31–2.12]	12; 1.68 [1.19–1.88]	NS
Stage C	14; 1.69 [1.28–1.99]	7; 1.62 [0.67–1.82]	NS

*IQR* Interquartile range, *NS* Non-significant. The *p-*value denotes the probability of the change in ST2 concentrations from baseline to follow-up examination for each group. Data are presented as median (interquartile range). * *p* < 0.05, ** *p* < 0.001

Galectin-3 and ST2 concentrations showed no differences between DMVD-stages and the control group neither at baseline or at follow-up examination.

### NT-proBNP and cTnI measurements

**Fig. 3:**
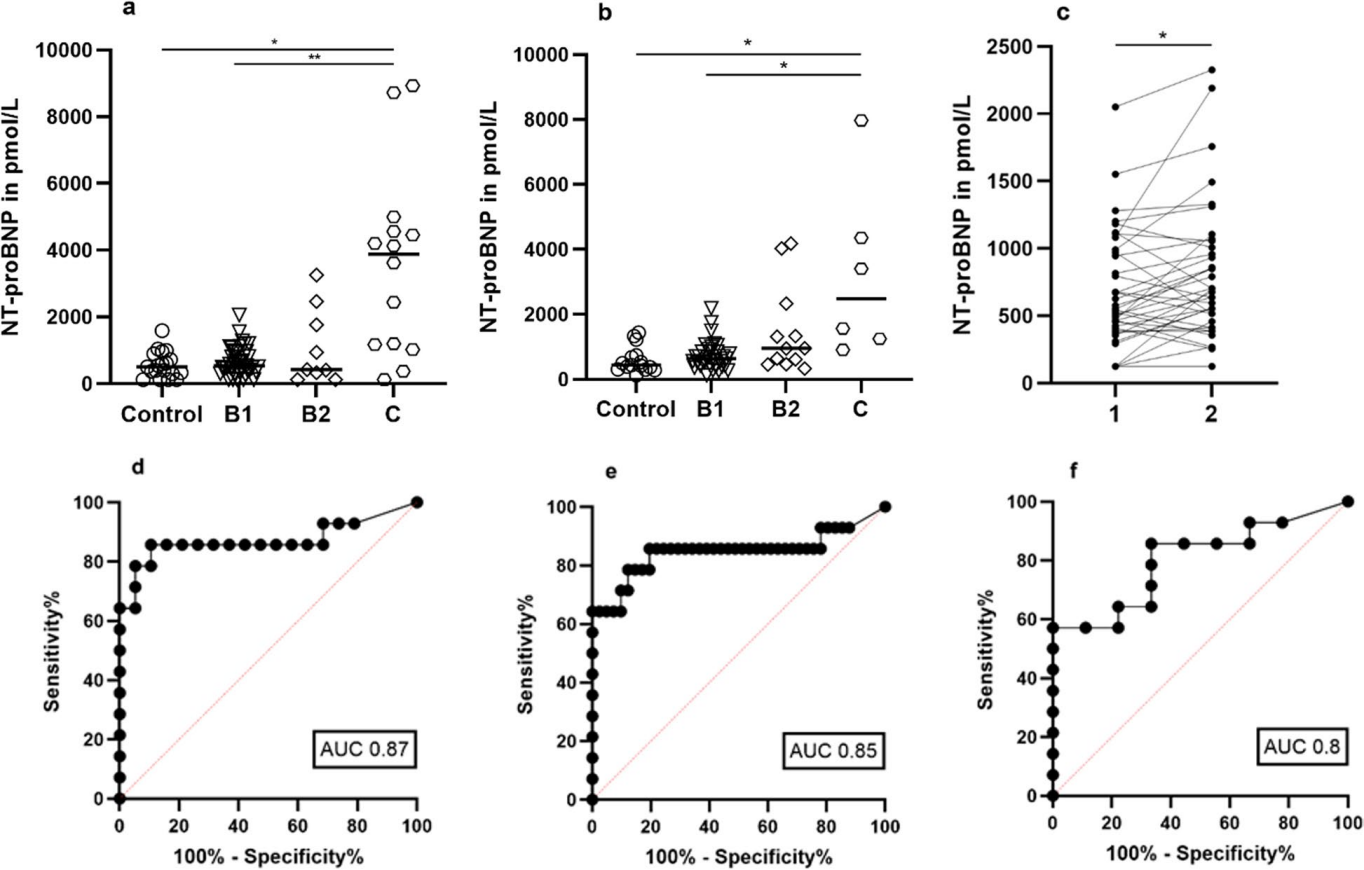
NT-proBNP concentrations in pmol/L for each dog in the respective groups accordingly at **a**) baseline and **b**) follow-up examination, median marked as a bar. **c**) Significant increase in NT-proBNP concentrations from baseline (**1**) to follow-up (**2**) occurred in stage B1 patients. ROC curves with AUC for comparing NT-proBNP between dogs with stage C and **d**) controls AUC 0,87 (95% CI, 0.72 to 1), **e**) stage B1 AUC 0.85 (95% CI, 0.69 to 1), and **f**) stage B2. * *p* < 0.05, **p < 0.001

Baseline NT-proBNP concentrations in all evaluated dogs with DMVD considered together (*n *= 64) ranged from 125 to 8930 (median [IQR] 670 [399–1196]) pmol/L, and remained similar (*p* = 0.5543) after six months follow-up (*n* = 51; 704 [465–1247] pmol/L). Median NT-proBNP concentrations (Table 5) increased according to disease progression through the clinical stages, except for the transition between stages B1 and B2 at baseline examination, where values decreased from stage B1 to stage B2. NT-proBNP concentrations of all DMVD patients considered together were significantly higher than in the healthy controls (*p* = 0.0341). Concentrations of healthy controls ranged from 125 to 1584 (median [IQR] 501 [298–898) pmol/L at baseline and from 125 to 1536 (median [IQR] 434 [301–735] pmol/L at second measurement. No significant differences between baseline and follow-up (*p* = 0.8895) were detected. Baseline concentrations of stage C patients were statistically different from controls (*p* = 0.0017) and stage B1 (*p* = 0.0006) (Fig. 3a), as were those at the follow-up examination (*p* = 0.01) and (*p* = 0.0045) (Fig. 3b). However, NT-proBNP concentrations were not able to differentiate individuals in earlier clinical stages from dogs in the control group. When comparing both evaluations performed, NT-proBNP concentrations increased significantly from the baseline to the follow- up examination in the group of stage B1 patients (*p* = 0.0113), see Fig. 3c and Table 5.

**Table 5:** NT-proBNP concentrations in pmol/L for the respective groups at baseline and follow-up examination

Groups	Baseline (*n*; median [IQR])	Follow-up (*n*; median [IQR])	*P*-value
Control	19; 501 [298–898]	15; 434 [301–735]	NS
Stage B1	41; 541 [389–955]	33; 636 [412–859]	0.0113*
Stage B2	9; 423 [332–1763]	12; 952 [537–1828]	NS
Stage C	14; 3876 [1192–4563]	6; 1247 [2483–4353]	NS

*IQR* Interquartile range, *NS* Non-significant. Data are presented as median (IQR). The *p*-value denotes the probability of the change in NT-proBNP concentrations from baseline to follow-up examination for each group. * *p *< 0.05

As pairwise comparisons revealed significant differences, ROC curve analyses were performed to differentiate dogs with stage C from all the other groups (Fig. 3d-f). Cut-off values were chosen based on the Youden’s index [57] for each baseline and follow-up examination (Table 6). With moderate strength (0.7 < AUC < 0.9), a cut-off value of 1005 pmol/L separated patients with stage C from controls and dogs with stage B1 at the baseline examination in their NT-proBNP concentrations. Furthermore, at the baseline examination, 2 of 14 stage C patients had values under this cut-off, and one of them was sampled for biomarker measurement after stabilization with an extensive initial therapy during hospitalization. On the other hand, the control group (*n* = 21) included 4 animals with values above 1005 pmol/L, of which 3 animals also had high values at follow-up. In the echocardiographic examination, however, no abnormalities were found that would have indicated heart disease. Similarly, 8 of 41 dogs in stage B1 had values above 1005 pmol/L. Two of these 8 animals were CKCS and one of them developed congestive heart failure (CHF) (stage C) about one year later. Of those 5 dogs in stage B1 that progressed to stage B2 at the follow-up examination, 3 had values above 1005 pmol/L at both evaluations. The same was true for the dog that progressed from stage B2 to stage C. At the second examination, lower cut-offs separating stage C from controls (821 pmol/L) and stage B1 (883 pmol/L) were detected. In both cases, sensitivity was higher (100%) and AUC values were highly accurate (0.9 < AUC < 1), but significance was reduced in comparison to the baseline evaluation (Table 6).

**Table 6:** Selected cut-offs for NT-proBNP concentrations based on the Youden index

**Investigation**	**Cut-off (pmol/L)**	**Sensitivity% **(**95% CI**)	**Specificity% (95% CI)**	**AUC (95% CI)**	***p*-value**
*Stage C compared with controls*					
Baseline	> 1005	85.71 (60.06–97.46)	89.47 (68.61–98.13)	0.87 (0.7–1)	0.0003
Follow-up	> 821	100 (60.97–100)	80 (54.81–92.95)	0.94 (0.85–1)	0.0018
*Stage C compared with stage B1*					
Baseline	> 1005	85.71 (60.06–97.46)	80.49 (65.99–89.77)	0.85 (0.69–1)	0.0001
Follow-up	> 883	100 (60.97–100)	75.76 (58.98–87.17)	0.93 (0.85–2)	0.0008
*Stage C compared with stage B2*					
Baseline	> 978.5	85.71 (60.06–97.46)	66.67 (35.42–87.94)	0.8 (0.62–0.98)	0.0167
Follow-up	> 1103	83.33 (43.65–99.25)	58.33 (31.95–80.67)	0.76 (0.53–0.99)	NS

This table shows selected cut-offs for NT-proBNP concentrations based on the best values for sensitivity and specificity to differentiate stage C from the other groups at baseline and follow-up examination, *CI* Confidence interval, *AUC* Area under the curve, *NS* Non-significant

A cut-off of 978.5 pmol/L separated patients with stage C from those with stage B2 at the first examination by means of their NT-proBNP concentrations; a higher cut-off 1103 pmol/L was detected at second examination. However, this second cut-off displayed a reduced sensitivity and specificity compared to that of the first examination and lacked significance (Table 6). Furthermore, at baseline examination, 4 of 9 animals in stage B2 had values above 978.5 pmol/L, one of which developed clinical symptoms and was categorized as stage C three months later. The other 3 animals maintained high values in a stable clinical state.

**Fig. 4:**
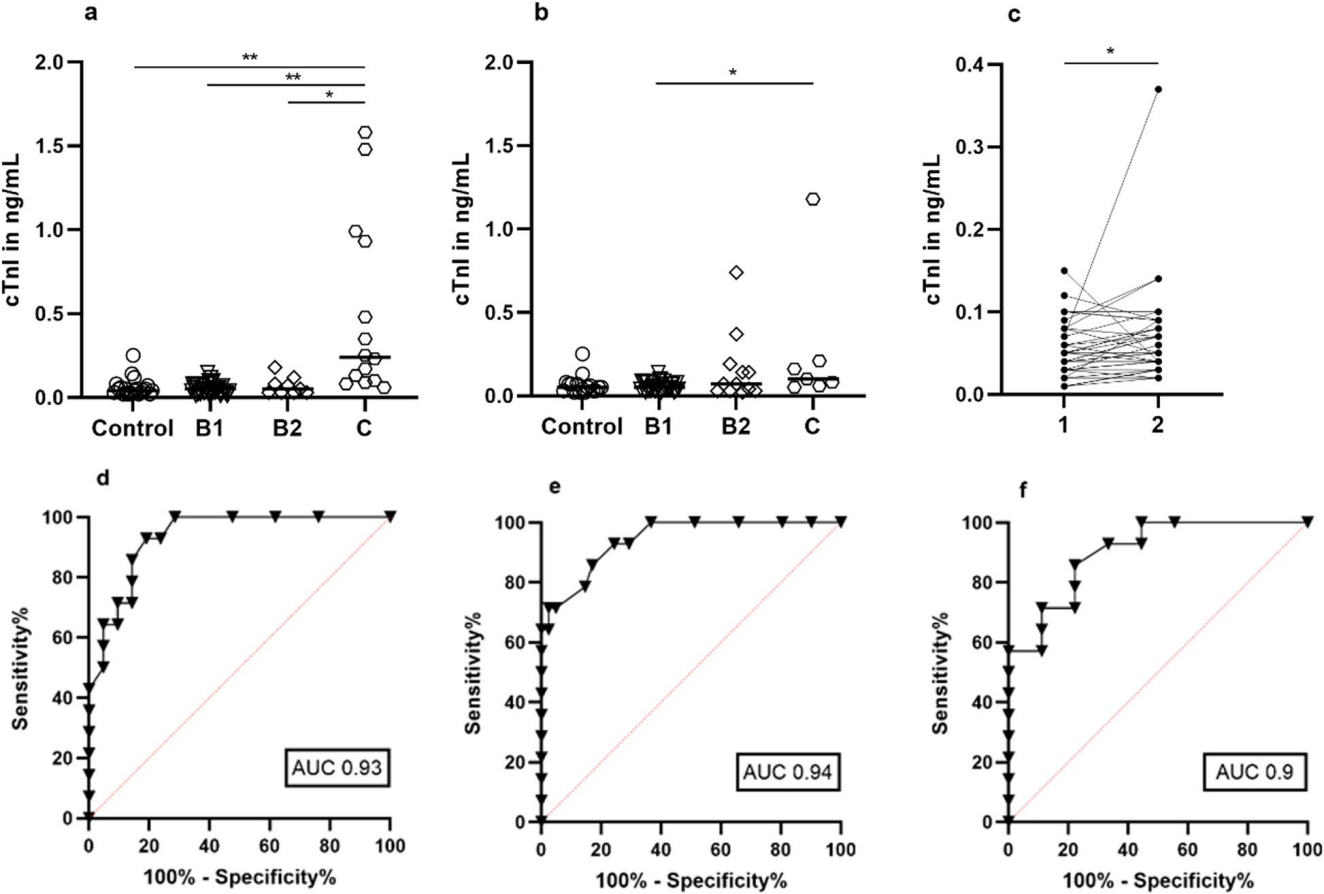
cTnI concentrations in ng/mL for each dog in the respective groups at **a**) baseline and **b**) follow-up examination, median marked as a bar. **c**) Significant increase in cTnI concentrations from baseline (**1**) to follow-up (**2**) occurred in stage B1 patients. ROC curves and AUC comparing cTnI between dogs with stage C and **d**) controls, **e**) stage B1, and **f**) stage B2. * *p* < 0.05, ** *p* < 0.001

As observed with NT-proBNP, cTnI concentrations were significantly different between stage C and healthy controls (*p* < 0.0001) or lower disease stages (Fig. 4a and b). Thus, the *p*-values for the comparison between stage C with stages B1 and B2 were < 0.0001 and 0.0078, respectively. CTnI concentrations at the first examination in all evaluated dogs with DMVD (*n *= 64) ranged from 0.01 to 1.58 (median [IQR] 0.06 [0.03–0.1]) ng/mL, and remained similar (*p* = 0.9822) after a six-month follow-up (*n* = 52; 0.06 [0.04–0.09] ng/mL). The median concentrations of the four groups at the baseline and follow-up examination are shown in Table 7. Concentrations in healthy controls ranged from 0.02 to 0.25 (median [IQR] 0.04 [0.03–0.06) ng/mL at baseline and from 0.02 to 0.25 (median [IQR] 0.05 [0.03–0.07] ng/mL at follow-up examination. No significant differences between baseline and follow-up (*p* = 0.5829) were detected. Baseline concentrations of stage C patients were statistically different from controls (*p* < 0.0001), stage B1 (*p* < 0.0001) and stage B2 (*p* = 0.0078) (Fig. 4a) as well as at follow-up examination from stage B1 (*p* = 0.0346) (Fig. 4b). Furthermore, an increase in cTnI concentrations from baseline to follow-up examination was significant in stage B1 (*p* = 0.0105) (Fig. 4c).

**Table 7:** cTnI concentrations in ng/mL for the respective groups at baseline and follow-up examination

Groups	Baseline (*n*; median [IQR])	Follow-up (*n*; median [IQR])	*P*-value
Controls	21; 0.04 [0.03–0.06]	17; 0.05 [0.03–0.07]	NS
Stage B1	41; 0.05 [0.03–0.07]	33; 0.05 [0.04–0.08]	0.0105*
Stage B2	9; 0.05 [0.03–0.08]	12; 0.07 [0.03–0.17]	NS
Stage C	14; 0.24 [0.1–0.93]	7; 0.1 [0.06–0.21]	NS

*IQR* Interquartile range, *NS* Non-significant. The *p*-value denotes the probability of the change in cTnl concentrations with a significant increase from baseline to follow-up examination for each group. * *p* < 0.05

The ROC curves for cTnI concentrations at baseline examination are shown in Fig. 4d-f. On the basis of the Youden`s index [57] and highly accurate AUC values (0.9 < AUC < 1), a cut-off value of 0.085 ng/mL for cTnl concentration differentiated patients with stage C from controls, and patients with stage B1 and stage B2 (Table 8). Furthermore, only 2 of 14 dogs in stage C had values under 0.085 ng/mL at the first examination. These 2 patients were treated with medication and were not presented with acute symptoms. While one of them was stable at follow-up examination, the other one increased above 0.085 ng/mL (0.1 ng/mL). The 2 dogs with the highest cTnl values (1.58 and 1.48 ng/mL) within stage C died before the follow-up appointment.

**Table 8:** Selected cut-off values for cTnI concentrations based on the Youden index

Investigation	Cut-off (ng/mL)	Sensitivity% (95% CI)	Specificity% (95% CI)	AUC (95% CI)	*p*-value
*Stage C compared with controls*					
Baseline	> 0.085	85.71(60.06–97.46)	85.71(65.36–95.02)	0.93(0.86–1)	< 0.0001
Follow Up	> 0.075	71.43(35.89–94.92)	82.35(58.97–93.81)	0.82(0.64–0.99)	0.0158
*Stage C compared with Stage B1*					
Baseline	> 0.085	92.86(68.53–99.63)	75.61 (60.66–86.17)	0.94(0.88–1)	< 0.0001
Follow Up	> 0.075	71.43(35.89–94.92)	69.70 (52.66–82.62)	0.83 (0.66–0.99)	0.0072
*Stage C compared with Stage B2*					
Baseline	> 0.085	85.71(60.06–97.46)	77.78(45.26–96.05)	0.9 (0.78–1)	0.0015
Follow-up	> 0.075	71.43(35.89–94.92)	58.33(31.95–80.67)	0.65 (0.41–0.9)	NS

This table shows selected cut-off values for cTnI concentrations based on the best values for sensitivity and specificity to differentiate Stage C from all other groups at baseline and follow-up examination, *CI* Confidence interval, *AUC* Area under the curve, *NS* Non-significant

On the other hand, 2 animals in the control group displayed values above 0.085 ng/mL, both of them showing similar values at the follow-up examination. At the first examination, 7 of 41 dogs in stage B1 had values above 0.085 ng/mL and 2 of the 3 oldest patients in stage B2 (> 12 years).

NT-proBNP and cTnI concentrations correlated with disease severity.

### Correlation between biomarkers, clinical variables and echocardiographic measurements

The Spearman correlation coefficient confirmed a good agreement between NT-proBNP and cTnI with (*r* = 0.56, *p* < 0.0001) at baseline and (*r *= 0.47, *p* < 0.0001) at follow-up examination. Concerning the other parameters, NT-proBNP concentrations correlated with LVIDDn (*r* = 0.46, *p* < 0.0001), age (*r* = 0.33, *p *= 0.0022), and LA/Ao (*r* = 0.29, *p* = 0.0094) at baseline examination. At follow-up examinations, NT-proBNP correlations were as follows: LVIDDn (*r* = 0.53, *p* < 0.0001), Galectin-3 (*r* = 0.36, *p* = 0.0029), age (*r* = 0.33, *p* = 0.0066), and LA/Ao (*r *= 0.28, *p* = 0.0404). CTnI concentrations correlated with age (*r* = 0.64, *p* < 0.0001), LA/Ao (*r* = 0.41, *p* = 0.0001), and LVIDDn (*r *= 0.21, *p* = 0.0499) at baseline examination. At follow-up examination, cTnI correlations were as follows: age (*r *= 0.63, *p* < 0.0001), LA/Ao (*r* = 0.25, *p* = 0.0404), Galectin-3 (*r* = 0.24, *p* = 0.045), and LVIDDn (*r* = 0.24, *p* = 0.0453). All correlations listed were significant and positively related. Most of them were of fair strength (< + 0.5/ – 0.5), some were poor (< + 0.3/—0.3) and some were moderate (+ 0.6/—0.6).

## Discussion

Biomarker testing is already one of the standards for monitoring heart disease in human medicine [25]. Natriuretic peptides are relevant for diagnosis and prognosis in HF patients, and biomarkers of myocardial injury (cTnI) and myocardial fibrosis (Galectin-3 and ST2) are used for risk stratification [58]. In veterinary medicine, the biomarkers NT-proBNP and cTnI are used for complementary diagnostic purposes [59]. Therefore, the current study aimed to investigate the usefulness of the proposed biomarkers Galectin-3 and ST2 to support diagnosis and staging of canine patients with DMVD, and to explore possible correlations with established cardiac biomarkers NT-proBNP and cTnI, as well as echocardiographic measurements.

A recently published study by Lee et al. (2021) evaluated Galectin-3 serum concentrations in healthy dogs, those with heart disease, and dogs with non-cardiac diseases (e.g., endocrine, neoplastic) [50]. They detected significantly higher concentrations of Galectin-3 in dogs with cardiac (1.12 ± 0.83 ng/mL) and non-cardiac diseases (2.27 ± 2.59 ng/mL) compared to the healthy group (0.64 ± 0.15 ng/mL). Further differentiation of the dogs with cardiac diseases revealed that there was no significant difference between the healthy group and the DMVD group, which is in line with the results of the current study. When subdividing patients into different types of cardiomyopathy, significantly higher Galectin-3 concentrations were detected in dogs with concentric cardiomyopathy compared to those with eccentric cardiomyopathy, such as DMVD. The finding that Galectin-3 was increased in dogs with concentric cardiac diseases could indicate that this biomarker might be more suitable for investigating other cardiac diseases than DMVD. Additionally, it is important to note that the Galectin-3 concentrations in dogs with non-cardiac diseases like endocrine or dermatologic diseases were even higher than in dogs with cardiac diseases [50]. This could indicate that Galectin-3 might be more responsive to non-cardiac diseases in dogs. Since dogs with relevant non-cardiac diseases were excluded from the present study, no information about the response to other diseases could be evaluated. Another important point to note regarding the group composition in the study by Lee et al. (2021) is that the healthy group consisted entirely of beagle dogs, while the other groups consisted of different breeds [50]. Since breed influences are known to exist for other biomarkers [60] and have not been investigated for Galectin-3, breed-specific differences cannot be ruled out as a reason for the measured differences from the current study and the study by Lee et al. (2021) [50].

Galectin-3 concentrations measured in the current study were not statistically different between healthy dogs (6380.50 [4462.49–7656.29] pg/mL) and DMVD patients (5069.60 [4334.51–7049.37] pg/mL) in total or between different DMVD stages. Thus, an increase in serum concentration with disease severity could not be confirmed [61]. In fact, Galectin-3 concentrations of dogs in stage B1 and the control group were even higher than those of dogs in stage B2 at baseline examination. This could be considered an effect of medication which was applied from stage B2 onwards. Results of the current study are in contrast to those from Sakarin et al. (2016) investigating both plasma Galectin-3 values and its expression in canine cardiac muscle in healthy dogs and DMVD patients [49]. They were able to detect a difference in median plasma Galectin-3 concentrations between controls (0.42 ng/mL) and patients with DMVD (1.49 ng/mL), but not among specific ACVIM stages. Additionally, histopathologic investigations in the study by Sakarin et al. (2016) revealed an increased Galectin-3 expression in DMVD and its positive correlation with areas of fibrosis [49]. However, since the histologic assessment was conducted in those dogs that were not tested for plasma Galectin-3, a direct correlation between Galectin-3 expression in the cardiac tissue samples and plasma level is difficult to establish. Further studies reported the presence of cardiac fibrosis in dogs [62, 63]. However, cardiac fibrosis might not represent a cardinal feature in canine DMVD [64] and its occurrence might be secondary [65]. When considering a reported role of Galectin-3 as fibrosis regulator and marker, it is plausible that pathologic differences lead to a higher magnitude of cardiac fibrosis in humans than in dogs, reducing the practicability of Galectin-3 for detection in dogs with DMVD. The usability of Galectin-3 and ST2 as appropriate predictive and prognostic biomarkers for the disease development or presence of fibrosis in canine DMVD should be further investigated.

Concerning ST2 measurements, our results are in line with those of Kim et al. (2018), in a study investigating ST2 concentrations alongside NT-proBNP in dogs with DMVD where no significant differences in ST2 measurements between patients and controls were detected [47]. In that mentioned study, dogs were divided into a healthy, asymptomatic, and symptomatic DMVD group with mean values of 2.050 ng/mL, 2.447 ng/mL, and 2.433 ng/mL. Overall, the ST2 median concentrations in the present study were lower than those detected by Kim et al. (2018) with 1.424 ng/mL for controls, 1.623 ng/mL for B1 and 1.339 ng/mL for B2 (representing asymptomatic patients), and 1.587 ng/mL for symptomatic patients allocated in stage C. However, in both studies similar slightly lower values were detected in healthy controls. Even though the same ELISA assay was used in both studies, a direct comparison of the results is restricted due to possible differences in serum samples processing, laboratory equipment, ambient temperature, as well as study-groups composition. When interpreting ST2 results, the specificity of the antibodies in the employed assay must also be kept in mind. The ELISA-kit employed detects soluble ST2 when it is not bound to its ligand IL-33 (MyBioSource, Inc., San Diego, CA, USA). Due to the lack of knowledge about the IL-33 concentration in the study population, it is possible that higher amounts of ST2 were released, but were already bound to its ligand and thus not detected by the test kit employed.

In the present study, an increase in ST2 values was detected in stage B1 when comparing the baseline to the follow-up examination. Dogs in that group also showed worsening in echocardiographic measurements, indicating that ST2 may be able to detect disease progression and could be applicable for monitoring DMVD patients over time [66]. However, a similar phenomenon was detected in the control group. The reason for the small but significant increase in the control group is not known. As the other biomarkers showed no differences in the control group, ST2 concentrations might possibly have been affected by technical methods or other non-evaluated occurrences. In human cardiac patients, serial measurements of ST2 can improve predicting the worsening of heart disease and death [67]. Patients with heart failure and values above the cut-off of ≥ 35 ng/mL do have a worse prognosis, rising linearly to ST2 serum elevation [68]. The same might be true for dogs, but further analyses would be needed to confirm or disprove this assumption. The large difference of ST2 concentrations in dogs compared to those in humans could be due to the fact that the abovementioned cut-off ≥ 35 ng/mL is based on the measurements with the high-sensitivity Presage ST2 assay (Critical Diagnostics, San Diego, CA, USA). In human medicine, this assay is accepted by the U.S. Food and Drug Administration (FDA) and is used for clinical purposes [69]. Other commercially available kits in human medicine, such as MBL ST2 ELISA Kit (Medical & Biological Laboratories, Woburn, MA, USA) and R&D DuoSet ELISA human ST2/IL-1 R4 (R&D Systems, Inc., Minneapolis, MN, USA), measure in comparable concentrations in humans as the assay used in the current study in dogs. Another study comparing ST2 concentrations of the three mentioned assays pointed out considerable differences between them [70]. These differences might occur due to different assay reagents [70], this pointing out that it is difficult to compare results measured with different assays. All assays used in humans detect ST2 in its unbound and bound form [69]. These findings suggest a need for a high-sensitivity assay in small animal medicine.

As Galectin-3 and ST2 are also markers of inflammation, this might indicate that they are not suitable for diagnosing and staging early stages of DMVD due to the lack of inflammatory processes during disease establishment [60]. Nonetheless, inflammation could be expected in dogs with severe disease (stages C and D), when the surface of the mitral valves suffers injury due to extensive reorganization processes or in rare cases of *chordae tendineae* rupture [71]. *Chordae tendineae* rupture in humans is still much more frequent than in dogs [72]. Considering the presence of secondary fibrosis and inflammation in already decompensated patients, Galectin-3 and ST2 could be useful in those severe cases. However, due to the design of the present study, the small sample size of stage C patients, and the absence of ruptured *chordae tendineae* in dogs evaluated, it is not possible to draw any conclusions regarding the potential usefulness of Galectin-3 and ST2 concentrations in severely ill canine patients with stages C and D. As a suggestion, future studies should focus on these severely ill dogs with attention to specific complications such as mitral flail or *chordae tendineae* rupture in echocardiographic examinations.

In the present study, no convincing correlations for the new biomarkers and DMVD in dogs were detected. A fair correlation [55] between Galectin-3 with both NT-proBNP (*r* = 0.36) and cTnI (*r* = 0.24) was detected at the follow-up examination, although no differentiation between the four groups in the means of Galectin-3 concentrations could be established. As the pairwise comparisons already showed an increase in NT-proBNP and cTnI with the disease stage, the Spearman correlation coefficient confirmed a moderate agreement (*r *= 0.56) of these two biomarkers. However, the best correlation was found for cTnI with age (*r* = 0.64).

NT-proBNP and cTnI concentrations increased with the progression of DMVD and thus were significantly higher in dogs with stage C than in asymptomatic dogs (B1 and B2) and in the control group. These findings are consistent with previous studies including dogs with and without medication [11, 15, 73–75]. Referring to the results of the ROC curves, despite slight differences between first and second evaluation, NT-proBNP and cTnI were very accurate to differentiate dogs with stage C from other groups. The cut-off value (883 pmol/L) for NT-proBNP at the follow-up even matches the reference value, of < 900 pmol/L provided by the performing laboratory IDEXX (IDEXX Laboratories) for dogs with a heart murmur but without symptoms. Treatment with diuretics for example can lead to a decrease in natriuretic peptide concentration [76]. This and the difference in group size might have contributed to the lower NT-proBNP concentrations in stage B2 than in stage B1 dogs in the present study. It should be noted that a direct comparison between the present and previous studies might be aggravated as previous studies predominantly included dogs with symptoms of CHF. In contrast, no differentiation of dogs with acute or past symptoms was made in the present study. On the other hand, the cut-off values for cTnI concentrations at baseline (0.08 ng/mL) and follow-up examination (0.075 ng/mL) exceeded the reference of < 0.06 ng/mL given by IDEXX (IDEXX Laboratories).

Several limitations were present in the current study. Due to the dependence on recruiting trial participants, animal numbers were unequal in the different study groups with an overrepresentation of stage B1. No separate consideration was made according to gender and the dogs enrolled were presented with a wide age range. The loss of some dogs at the follow-up examination, might have decreased statistical significance. In addition, the frequency of the examinations and the timespan between them were insufficient to draw conclusions about the disease development. Especially for patients in stage C, the timespan to follow-up examination was too long and thus half of the group was lost to cardiac death. Moreover, no pre-selection was made in terms of medication, as some patients already received medication and others did not. In addition to this, the stage C group was composed of dogs with and without current symptoms of CHF, resulting in a somewhat heterogeneous group. Therefore, no conclusions concerning the predictive benefit could be drawn from this study. Focusing on dogs with severe DMVD and a further distinction between patients with acute and recurrent symptoms might be advisable for future studies. Another limitation of the present study was the moderate inter-assay CV, which limits reproducibility and mitigates the interpretation of the results.

## Conclusions

In conclusion, the results of the present study provide evidence that the new biomarkers Galectin-3 and ST2 are both measurable biomarkers in the canine patient. However, neither for Galectin-3 nor for ST2, significant differences in serum concentrations between the different dogs with or without DMVD, and across clinical stages of DMVD were found. Thus, it might indicate that they are unsuitable for diagnosing and staging canine DMVD patients. On the other hand, as results of the current study suggest, serial measurements of ST2 might be helpful for monitoring the disease progression, response to treatment, and could be a valid approach in conjunction to survival analysis. As the results concerning Galectin-3 concentrations from the current study and former studies are discordant, the usefulness of Galectin-3 in heart disease should be further investigated. In agreement with previous reports, the diagnostic value of NT-proBNP and cTnI for detecting dogs with severe DMVD was confirmed. Reliable differentiation between the different preclinical stages of DMVD was not possible in our study with any of the examined biomarkers. Furthermore, the identification of other meaningful cardiac biomarkers for canine cardiac patients and the development of a multimarker strategy for diagnosing and staging DMVD, and individual treatment modifications in dogs are worth pursuing and should be continued, though our results indicate that ST2 and Galectin-3 may be of limited value to achieve this goal.

## Data Availability

The datasets used and analyzed during the current study are available from the corresponding author on reasonable request.

## Abbreviations

ACEI: Angiotensin-Converting Enzyme Inhibitor
ACVIM: American College of Veterinary Internal Medicine
AUC: Area under the curve
BUN: Blood Urea Nitrogen
CHF: Congestive heart failure
CKCS: Cavalier King Charles Spaniel
CREA: Creatinin
cTnI: Cardiac Troponin I
CV: Coefficient of variation
DMVD: Degenerative mitral valve disease
f: Female
fs: Female spayed
HF: Heart failure
IL-33: Interleukin-33
IQR: Interquartile range
LA/Ao: Ratio left atrium to aortic root
LVIDDn: Left ventricular internal diastolic diameter normalized
m: Male
mn: Male neutered
NT-proBNP: N-terminal-prohormone-B-type natriuretic peptide
ROC: Receiver-operating characteristic
SD: Standard deviation
ST2: Interleukin-1 receptor-like 1 protein
ST2L: Transmembrane interleukin-1 receptor-like 1 protein
sST2: Soluble interleukin-1 receptor-like 1 protein
